# Criterion validity of the Ekblom-Bak and the Åstrand submaximal test in an elderly population

**DOI:** 10.1007/s00421-019-04275-7

**Published:** 2019-12-09

**Authors:** Daniel Väisänen, Örjan Ekblom, Elin Ekblom-Bak, Eva Andersson, Jonna Nilsson, Maria Ekblom

**Affiliations:** 1 Åstrand Laboratory of Work Physiology, The Swedish School of Sport and Health Sciences, Box 5626, 114 86 Stockholm, Sweden; 2 Department of Neuroscience, Karolinska Institute, 171 77 Stockholm, Sweden; 3 Aging Research Center, Karolinska Institute, Stockholm University, 171 77 Stockholm, Sweden

**Keywords:** Cardiorespiratory fitness, Elderly, Oxygen uptake, Submaximal test, Validity, Public health

## Abstract

**Purpose:**

The aim of this study was to validate the submaximal Ekblom-Bak test (EB-test) and the Åstrand test (Å-test) for an elderly population.

**Methods:**

Participants (*n* = 104), aged 65–75 years, completed a submaximal aerobic test on a cycle ergometer followed by an individually adjusted indirect calorimetry VO_2_max test on a treadmill. The HR from the submaximal test was used to estimate VO_2_max using both the EB-test and Å-test equations.

**Results:**

The correlation between measured and estimated VO_2_max using the EB method and Å method in women was *r* = 0.64 and *r* = 0.58, respectively and in men *r* = 0.44 and *r* = 0.44, respectively. In women, the mean difference between estimated and measured VO_2_max was − 0.02 L min^−1^ (95% CI − 0.08 to 0.04) for the EB method and − 0.12 L min^−1^ (95% CI − 0.22 to − 0.02) for the Å method. Corresponding values for men were 0.05 L min^−1^ (95% CI − 0.04 to 0.14) and − 0.28 L min^−1^ (95% CI − 0.42 to − 0.14), respectively. However, the EB method was found to overestimate VO_2_max in men with low fitness and the Å method was found to underestimate VO_2_max in both women and men. For women, the coefficient of variance was 11.1%, when using the EB method and 19.8% when using the Å method. Corresponding values for men were 11.6% and 18.9%, respectively.

**Conclusion:**

The submaximal EB-test is valid for estimating VO_2_max in elderly women, but not in all elderly men. The Å-test is not valid for estimating VO_2_max in the elderly.

**Electronic supplementary material:**

The online version of this article (10.1007/s00421-019-04275-7) contains supplementary material, which is available to authorized users.

## Introduction

Cardiorespiratory fitness (CRF) is established as a strong predictor of health (Kodama et al. 2009; Harber et al. 2017). A single measurement of CRF is a stronger predictor for mortality than high blood pressure, smoking, obesity and type 2 diabetes (Myers et al. 2002). CRF typically decreases with age (Betik and Hepple 2008); the rate of decline accelerates at 45 years and is even faster at 65 years (Jackson et al. 2009). Decreased CRF in the elderly can significantly impair functional capacity in everyday life and increase the risk of cardiovascular mortality (Kokkinos et al. 2010). From a health perspective, it is therefore particularly useful to monitor CRF in the elderly.

The maximal oxygen uptake (VO_2_max) test is the gold standard (Fletcher et al. 2001) for measuring CRF. Performing a VO_2_max test is time consuming and requires expertise and expensive ventilatory gas-exchange equipment. The test also requires the participant to perform a maximal effort that can be intimidating for some parts of the population. It is especially, challenging for an elderly population prone to abnormal gait (Mahlknecht et al. 2013), impaired balance (Lin and Bhattacharyya 2012), and muscular weakness (Julius et al. 1967). In addition, the elderly are often more apprehensive about performing maximal effort than younger age groups. Since CRF is such an important predictor of health outcomes, increasing its availability may enable identification of elderly individuals with low VO_2_max in need of medical care or lifestyle interventions. The American Heart Association has stated that CRF should be used as a clinical evaluation tool (Ross et al. 2016).

Submaximal tests estimate VO_2_max based on heart rate response at one or more submaximal work rates (Noonan and Dean 2000). The Åstrand test (Å-test) (Astrand and Ryhming 1954; Astrand 1960) is one of the most commonly used submaximal cycle ergometer tests and utilizes the heart rate response to one submaximal work rate. This test has been validated for a population up to 65 years. The validity of this method for individuals older than 65 years is to a large extent unknown.

Another predictive submaximal VO_2_max ergometer cycle test is the Ekblom-Bak (EB) test (Ekblom-Bak et al. 2014; Bjorkman et al. 2016). The EB-test consists of exercise at one standardized, low work rate followed by a higher, individually set work rate. This test has been developed and validated in a mixed sample of men and women (aged 20–86 years), with a wide range of VO_2_max (ranging from 1.33 to 5.97 L min^−1^). This test has shown reasonably strong validity (Ekblom-Bak et al. 2014; Bjorkman et al. 2016) with a coefficient of variation (CV) of 9.2% and 8.4% for women and men, respectively and a standard error of the estimate (SEE) of 0.24 L min^−1^ and 0.31 L min^−1^ for women and men, respectively.

Since neither tests has been validated for use in an elderly population of men nor women with a lower VO_2_max, the aim of this study was to validate the submaximal EB-test and Å-test in an elderly (> 65 years) population, using directly measured VO_2_max, as a reference. Based on previous research, we speculated that both the EB-test and the Å-test would give valid estimates of VO_2_max in an elderly population.

## Methods

### Participants

Participants were recruited through local newspapers and flyers. Exclusion criteria were severe joint problems, very high blood pressure, or other cardiovascular problems, psychiatric illness or neurological disease. Of the initially screened 170 volunteers, 120 performed the required maximal and submaximal tests for the present study. After being assessed for the VO_2_max criteria and the submaximal heart rate criteria for the Å method, a final sample of 104 participants was included (52 women and 52 men, age range 65–75 years with a mean age of 70.6 ± 2.9 years). Prior to undertaking the physical tests, participants answered a single item categorical answer mode questionnaire (Olsson et al. 2016), where they self-rated moderate to vigorous physical activity.

All participants visited the Åstrand Laboratory at the Swedish School of Sport and Health Sciences on one occasion to perform the submaximal and maximal testing. Test duration was approximately 60 min. Each participant provided written consent before the start of the tests. The present study is a part of a larger study, which was approved by the Stockholm ethical committee (2017/1115-31/4). Table 1 shows participant characteristics.

**Table 1:** Anthropometry and physiological characteristics of the study sample, mean ± standard deviation

	Age (years)	BMI	Body mass (kg)	Height (cm)	Measured VO_2_max (L min^−1^)	Measured VO_2_max (mL kg^−1^ min^−1^)	HRmax (beats/min)
All (*n* = 104)	70.6 ± 2.9	25.5 ± 3.2	75.4 ± 12.8	171.5 ± 8.9	2.36 ± 0.55	31.4 ± 5.3	165 ± 12
Women (*n* = 52)	70.6 ± 2.9	24.6 ± 3.3	66.6 ± 9.9	164.5 ± 5.1	1.89 ± 0.22	28.8 ± 4.6	167 ± 10
Men (*n* = 52)	70.5 ± 2.9	26.4 ± 2.8	83.7 ± 9.2	178.0 ± 6.4	2.82 ± 0.34	34.0 ± 4.6	162 ± 13

### Submaximal and maximal aerobic tests

Participants were instructed to abstain from eating 90 min prior and not to consume caffeine or nicotine 2 hours prior to testing. Furthermore, they were instructed not to perform any heavy or prolonged physical activity the day before or on the day of the test. The test started with participants resting in a seated position for 15 min, where they received oral information about the test procedure. Instructions were also given on how to use Borg´s scale of perceived exertion (Borg 1970). Participants were equipped with a heart rate (HR) monitor (Polar model H7, Kempele, Finland) and watch (Polar model M400, Kempele, Finland). The test was initiated at resting heart rate.

### Submaximal test

The submaximal test was performed on a mechanically braked cycle ergometer (Monark model 828E, Varberg, Sweden). Participants were instructed to pedal at a cadence of 60 RPM and not speak or adjust position for the duration of the test. Total duration of the test was 8 min, with the initial 4 min performed at a fixed work rate of 0.5 kilo pounds (kp), directly followed by 4 min at a higher individualized work rate that varied between 1–3 kp. The individualized work rate was subjectively chosen by the test leader with regard to gender, body size, training background, and training status. Mean HR was recorded for the final minute of each work rate (calculated as the average of the heart rate recorded at 3:15, 3:30, 3:45, and 4:00). At the high work rate, if the Borg RPE was lower than 12 after the first minute, the load was further increased and the test duration was increased by 1 min.

### Maximal test

A maximal incremental treadmill test was performed directly following the submaximal test. A maximal test uses the directly measured gas-exchange during an incremental effort to assess the gas-exchange threshold and hence the oxygen consumption (VO_2_max). To measure gas-exchange (O_2_ and CO_2_), a mask with a flow meter connected to a gas analyzer (Jaeger Oxycon Pro, Hoechberg, Germany) was used. Flow meter volume and gas were calibrated prior to each test using a precision gas mixture (15.00 ± 0.01% O_2_ and 6.00 ± 0.01% CO_2_, Air Liquid, Kungsängen, Sweden) and ambient indoor air. All participants wore a safety harness attached to the roof when performing the maximal treadmill test.

Before the maximal test, participants were allowed a short rest (~ 2 min) and a familiarization/warmup session (~ 1–2 min) followed by another short rest (~ 1–2 min). When performing the familiarization/warmup session, participants initially walked at an individualized comfortable speed and 1° inclination and progressed to an individualized higher speed and incline. The maximal incremental VO_2_max test started at a comfortable pace and 1° inclination, most times at a walking speed of 3.5–5.0 km/h. The protocol for each participant was individually set, with the aim of reaching respiratory exchange ratio (RER) 1.0 at 5–6 min and RER 1.1 at 6–8 min. A few participants were unable to run due to previous injuries and instead walked at a moderate speed and steep inclination. At the end of the maximal incremental test, speed and inclination were increased more frequently to ensure a maximal plateau of oxygen consumption (leveling off) was reached. Criteria for an approved test were: a leveling off in VO_2_ despite an increase in speed or incline, a RER ≥ 1.1, RPE ≥ 17, or test duration ≥ 5 min. When the plateau criteria and two of the remaining three criteria were met, the test was accepted as a VO_2_max. A small coefficient of variation (2.7%) between a first and second test using the same protocol as mentioned above has previously been reported (Howley et al. 1995; Ekblom-Bak et al. 2014).

### Data analysis

The EB-test calculations estimated VO_2_max (in L min^−1^) using the gender-specific equations (equation for women: ln VO_2_max = 1.84390 − 0.00673 (age) − 0.62578 (ΔHR/ΔPO) + 0.00175 (ΔPO) − 0.00471 (HR at standard work rate). Equation for men: ln VO_2_max = 2.04900 − 0.00858 (age) − 0.90742 (ΔHR/ΔPO) + 0.00178 (ΔPO) − 0.00290 (HR at standard work rate)) (Bjorkman et al. 2016). The equations uses the increase in heart rate (HR) in relation to the increase in work rate (PO), sex, age, and the HR at the lower and higher work rates. For the Å method, HR was taken from the higher work rate of the EB-test. The Å-test calculations estimated VO_2_max using the Åstrand nomogram and the Åstrand extrapolated age correction factors (with a decreasing factor from 65 years of 0.006 per year).

For relative VO_2_max, the absolute L min^−1^ was divided with body weight and multiplied by 1000 to get ml kg^−1^ min^−1^. Low fit was seen as the quartile with the lowest measured VO_2_max in women and men, respectively. High fit was seen as the quartile with the highest measured VO_2_max in women and men, respectively.

### Statistical analysis

SPSS Inc (Chicago, III, US) was used for all statistical analyses. Descriptive data are presented as mean ± SD (range). A Shapiro–Wilk test was used to determine normal distribution, which was present for all tested parameters.

Pearson’s correlation coefficient (*r*) was calculated between the variables estimated from the submaximal test and directly measured during the maximal test. Paired Student’s *t* tests were used to determine differences between measured and estimated VO_2_max. To determine whether validity was different for different fitness levels, HR levels, or self-reported physical activity levels, Pearson’s or Spearman’s correlation coefficients were calculated between the difference of estimated and measured VO_2_max and VO_2_max, maximal heart rate, and self-rated physical activity. We regarded the Pearson’s *r* and Spearman’s *ρ* as weak (< 0.10), modest (0.1–0.3), moderate (0.3–0.5), strong (0.5–0.8), or very strong (0.8–1.0). Standard errors of the estimate (SEE) were derived from a linear regression model to show the variation around the regression line. To determine the variation in relation to its mean, we used coefficient of variation (CV), which was calculated using the SD of the differences between measured and estimated VO_2_max divided by the mean of the two methods. 95% confidence intervals (95% CI) were calculated for the difference between estimated and measured VO_2_max. Limits of Agreement (LoA) was calculated using the equation: mean of the difference between estimated and measured VO_2_max ± 1.96 multiplied by the SD of difference between the two methods. LoA is expected to include 95% of the differences between the two measurement methods. Significance level was set at *p* < 0.05 and we regarded 0.05 ≥ *p* < 0.1 as a trend (Curran-Everett and Benos 2004).

## Results

Mean measured VO_2_max in women was 1.89 L min^−1^ ± 0.22 and mean estimated VO_2_max was 1.88 ± 0.3 L min^−1^, using the EB method and 1.76 L min^−1^ ± 0.44, using the Å method. In men, mean measured VO_2_max was 2.83 L min^−1^ ± 0.4 and mean estimated VO_2_max was 2.88 ± 0.3, using the EB method and 2.54 L min^−1^ ± 0.55, using the Å method.

**Fig. 1:**
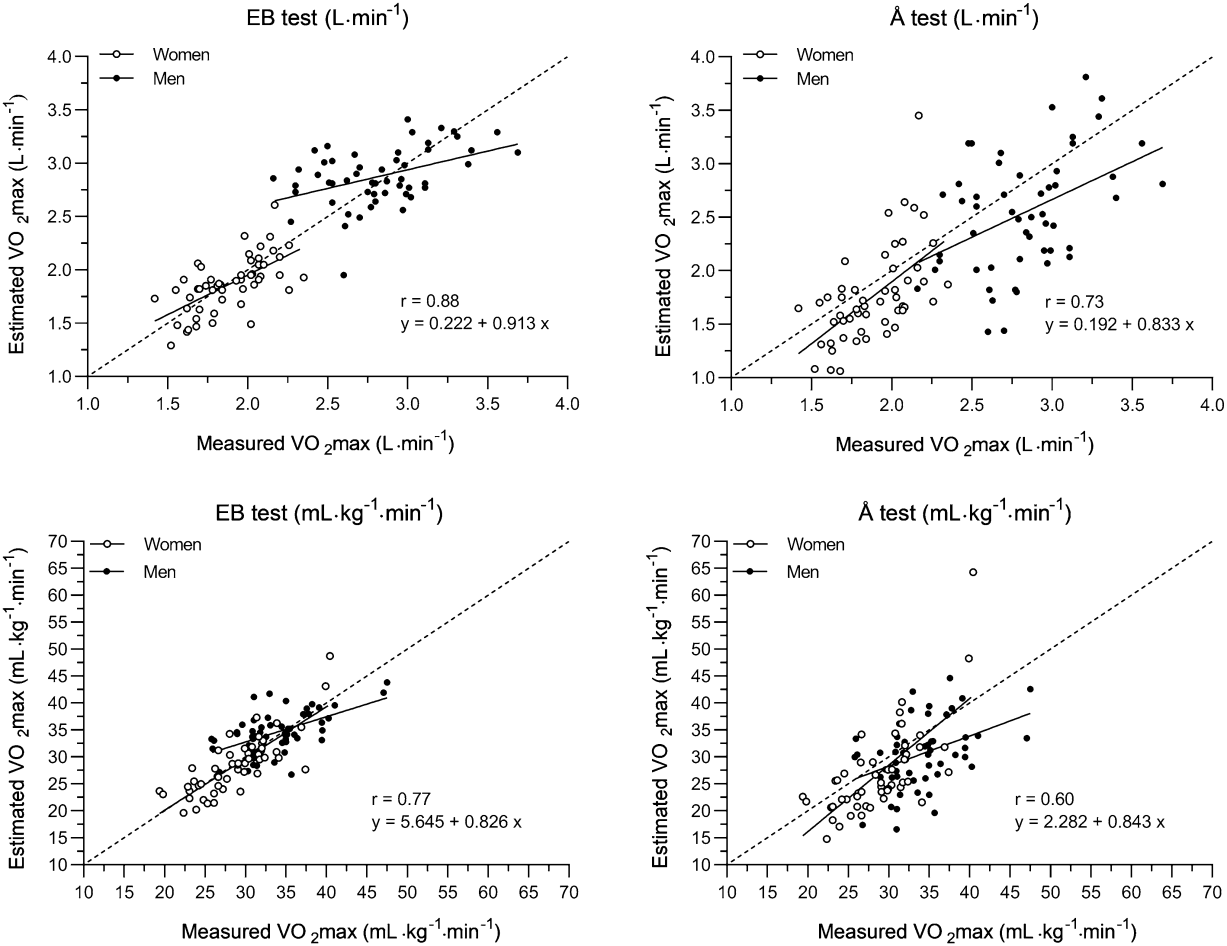
The submaximal EB method and the Å method vs. absolute (L min^−1^) and relative (ml kg^−1^ min^−1^) measured VO_2_max. The correlation coefficients and equations in the figure are for both genders together. Correlation coefficient, *r*, for absolute estimated VO_2_max in women was 0.64 (EB method) and 0.55 (Å method). Correlation coefficient, *r*, for absolute estimated VO_2_max in men was 0.44 (EB method and Å method). Correlation coefficient, *r*, for relative estimated VO_2_max in women was 0.81 (EB method) and 0.70 (Å method). Correlation coefficient, *r*, for relative estimated VO_2_max in men was 0.56 (EB method) and 0.49 (Å method)

There were no significant differences between estimated and measured VO_2_max in neither women nor men for the EB method. However, the Å method significantly underestimated women’s fitness by − 0.12 L min^−1^ (95% CI − 0.22 to − 0.02) and men’s fitness by − 0.28 L min^−1^ (95% CI − 0.42 to − 0.14) (Table 2 and Fig. 1). LoA for women was − 0.43–0.39 L min^−1^, when using the EB method and − 0.83–0.59 L min^−1^, when using the Å method. Corresponding values for men were − 0.60–0.70 L min^−1^ and − 1.28–0.71 L min^−1^, respectively (see online resource 1 to 4).

**Table 2:** Validity of the EB-test and the Å-test, absolute VO_2_max, mean ± standard deviation

	Measured VO_2_max (L min^−1^) mean ± SD	Estimated VO_2_max mean ± SD (L min^−1^)		Difference estimated vs. measured VO_2_max (L min^−1^) mean (95% CI)		Correlation coefficient (*r*)		Coefficient of variation (%)		SEE (L min^−1^)	
		EB-test	Å-test	EB-test	Å-test	EB-test	Å-test	EB-test	Å-test	EB-test	Å-test
All (*n* = 104)	2.36 ± 0.55	2.37 ± 0.57	2.15 ± 0.63	0.02 (− 0.03 to 0.07)	− 0.20 (− 0.29 to 0.12)	0.88**	0.73**	11.7	19.8	0.27	0.44
Women (*n* = 52)	1.89 ± 0.22	1.87 ± 0.26	1.76 ± 0.44	− 0.02 (− 0.08 to 0.04)	− 0.12 (− 0.22 to − 0.02)	0.64**	0.58**	11.1	19.8	0.20	0.36
Quartiles according to absolute VO_2_max level (L min^−1^)											
Q1 (*n* = 13)	1.61 ± 0.08	1.64 ± 0.22	1.45 ± 0.28	0.03 (− 0.10 to 0.17)	− 0.15 (− 0.33 to 0.02)	0.16	0.04	13.9	18.8	0.23	0.29
Q2 (*n* = 13)	1.78 ± 0.05	1.79 ± 0.14	1.59 ± 0.21	0.01 (− 0.08 to 0.11)	− 0.18 (− 0.32 to − 0.05)	− 0.07	− 0.16	8.8	13.2	0.15	0.22
Q3 (*n* = 13)	2.00 ± 0.04	1.94 ± 0.21	1.87 ± 0.35	− 0.06 (− 0.19 to 0.07)	− 0.13 (− 0.33 to 0.08)	0.15	0.21	10.6	17.7	0.22	0.36
Q4 (*n* = 13)	2.16 ± 0.09	2.10 ± 0.21	2.14 ± 0.53	− 0.06 (− 0.21 to 0.08)	− 0.02 (− 0.35 to 0.30)	− 0.11	0.23	11.2	24.9	0.22	0.56
Men (*n* = 52)	2.82 ± 0.34	2.88 ± 0.27	2.54 ± 0.55	0.05 (− 0.04 to 0.14)	− 0.28 (− 0.42 to − 0.14)	0.44**	0.44**	11.6	18.9	0.25	0.50
Quartiles according to absolute VO_2_max level (L min^−1^)											
Q1 (*n* = 13)	2.41 ± 0.12	2.86 ± 0.20	2.48 ± 0.45	0.46 (0.35 to 0.57)	0.07 (− 0.16 to 0.31)	0.33	0.59	7.4	16.0	0.19	0.38
Q2 (*n* = 14)	2.71 ± 0.07	2.67 ± 0.28	2.21 ± 0.57	− 0.03 (− 0.18 to 0.12)	− 0.50 (− 0.82 to − 0.18)	0.38	0.44	9.8	22.7	0.27	0.57
Q3 (*n* = 12)	2.94 ± 0.06	2.89 ± 0.22	2.50 ± 0.39	− 0.05 (− 0.19 to 0.09)	− 0.44 (− 0.68 to − 0.20)	0.11	0.21	7.6	13.8	0.23	0.39
Q4 (*n* = 13)	3.26 ± 0.21	3.10 ± 0.22	3.00 ± 0.50	− 0.17 (− 0.31 to − 0.02)	− 0.51 (− 0.57 to 0.04)	0.34	0.39	7.7	16.3	0.22	0.51

Quartiles in L min^−1^, women; < 1.69, 1.69–1.89, 1.89–2.07, 2.07 >; men; < 2.55, 2.55–2.80, 2.80–3.02, 3.02 >

*p < 0.05, **p < 0.01

CV was somewhat lower for the EB method compared to the Å method in both women (11.1% vs. 19.8%) and men (11.6% vs. 18.9%), accompanied with a smaller SEE in both women (0.20 L min^−1^ vs. 0.36 L min^−1^) and men (0.25 L min^−1^ vs. 0.50 L min^−1^) (Table 2).

Similar tendencies were found for relative estimated VO_2_max (ml kg^−1^ min^−1^) as for estimated absolute VO_2_max (L min^−1^) (Table 3). The EB method showed no bias for women (− 0.02 ml kg^−1^ min^−1^, 95% CI − 0.08 to 0.04) or men (0.05 ml kg^−1^ min^−1^, 95% CI − 0.04 to 0.14), while the Å method significantly underestimated women (− 0.12 ml kg^−1^ min^−1^, 95% CI − 0.22 to − 0.02) and men (− 0.28 ml kg^−1^ min^−1^, 95% CI − 0.42 to − 0.14). CV for the EB method and the Å method was 11.1% vs. 19.8% for women and 11.6% vs. 18.9% for men, respectively.

**Table 3:** Validity of the EB-test and the Å-test, Relative VO_2_max, mean ± standard deviation

	Measured VO_2_max (mL kg^−1^ min^−1^) mean ± SD	Estimated VO_2_max (mL kg^−1^ min^−1^) mean ± SD		Difference estimated vs. measured VO_2_max (mL kg^−1^ min^−1^) mean (95% CI)		Correlation coefficient (*r*)		Coefficient of variation (%)		SEE (mL kg^−1^ min^−1^)	
		EB-test	Å-test	EB-test	Å-test	EB-test	Å-test	EB-test	Å-test	EB-test	Å-test
All (*n* = 104)	31.4 ± 5.3	31.6 ± 5.6	28.8 ± 7.5	0.19 (− 0.53 to 0.91)	− 2.66 (− 3.83 to − 1.5)	0.77*	0.60*	11.7	20.1	3.59	6.02
Women (*n* = 52)	28.8 ± 4.6	28.6 ± 5.5	27.0 ± 8.2	− 0.25 (− 1.16 to 0.66)	− 1.80 (− 3.46 to − 0.15)	0.81*	0.70*	11.4	21.3	3.30	5.89
Quartiles according to absolute VO_2_max level (L min^−1^)											
Q1 (*n* = 13)	26.5 ± 4.9	26.8 ± 4.6	23.7 ± 4.7	0.30 (− 1.87 to 2.46)	− 2.89 (− 5.81 to 0.02)	0.72**	0.49	13.4	19.2	3.33	4.24
Q2 (*n* = 13)	27.5 ± 3.3	27.7 ± 4.7	24.8 ± 5.6	0.28 (− 1.22 to 1.79)	− 2.65 (− 4.82 to − 0.47)	0.86**	0.80**	9.0	13.7	2.49	3.54
Q3 (*n* = 13)	29.7 ± 4.5	28.7 ± 4.2	27.6 ± 5.1	− 1.07 (− 3.15 to 1.02)	− 2.14 (− 5.25 to 0.97)	0.69**	0.44	11.8	18.0	3.20	4.80
Q4 (*n* = 13)	31.5 ± 4.4	31.0 ± 7.6	31.9 ± 12.6	− 0.51 (− 2.70 to 1.67)	0.46 (− 4.92 to 5.84)	0.96**	0.89**	11.6	28.1	2.28	5.90
Men (*n* = 52)	34.0 ± 4.6	34.7 ± 3.9	30.5 ± 6.3	0.63 (− 0.50 to 1.76)	− 3.51 (− 5.21 to − 1.81)	0.56*	0.49*	11.8	18.9	3.27	5.82
Quartiles according to absolute VO_2_max level (L min^−1^)											
Q1 (*n* = 13)	30.2 ± 3.4	35.8 ± 3.2	30.8 ± 4.2	5.62 (4.17 to 7.08)	0.58 (− 2.41 to 3.57)	0.73**	0.16	7.3	16.2	2.28	4.35
Q2 (*n* = 14)	33.8 ± 3.4	33.3 ± 4.1	27.6 ± 7.7	− 0.50 (− 2.49 to 1.50)	− 6.18 (− 10.16 to − 2.19)	0.59*	0.44	10.3	22.5	3.44	7.19
Q3 (*n* = 12)	34.7 ± 3.1	34.1 ± 3.2	29.5 ± 4.6	− 0.67 (− 2.27 to 0.93)	− 5.22 (− 7.96 to − 2.49)	0.68*	0.43	7.3	13.4	2.46	4.36
Q4 (*n* = 13)	38.4 ± 5.4	35.5 ± 4.7	34.3 ± 6.5	− 1.96 (− 3.67 to − 0.25)	− 3.15 (− 6.8 to 0.46)	0.85**	0.50	7.8	16.7	2.58	5.84

Quartiles in L min^−1^, women; < 1.69, 1.69–1.89, 1.89–2.07, 2.07 > ; men; < 2.55, 2.55–2.80, 2.80–3.02, 3.02 >

*p < 0.05, **p < 0.01

In women, the estimated error (difference between estimated and measured VO_2_max) for the EB method displayed a trend toward being associated with VO_2_max level (*p* = 0.051, *r* = − 0.27) and self-rated physical activity level (*p* = 0.059, *ρ* = 0.26). It was also significantly associated with maximal HR level (*p* < 0.01, *r* = − 0.60). In men, the estimated error was associated with VO_2_max level (*p* < 0.01, *r* = − 0.67) and maximal HR level (*p* < 0.05, *r* = − 0.32), but not self-rated physical activity level (*p* = 0.22, *ρ* = − 0.17). Estimated error for the Å method in women was not associated with VO_2_max level (*p* = 0.50, *r* = − 0.10), but was with maximal HR level (*p* < 0.01, *r* = − 0.654) and self-rated physical activity level (*p* < 0.05, *ρ* = 0.28). In men, estimated error was not associated with VO_2_max level (*p* = 0.17, *r* = − 0.19) or self-rated physical activity level (*p* > 0.05, *ρ* = 0.05), but was associated with maximal HR level (*p* < 0.01, *r* = − 0.60).

## Discussion

The main finding was that there was good agreement between measured and estimated VO_2_max when using the EB method in a population of elderly men and women. In addition, there was good agreement between estimated, using the EB method, and measured VO_2_max over the full VO_2_max spectrum for women. Low fit men were overestimated and high fit men were partly underestimated using the EB method. The Å method significantly underestimated VO_2_max in both women and men. Precision expressed as CV and SEE for the EB method was almost half that of the Å method in both men and women.

No previous studies have validated the EB or the Å-test in a large elderly population (> 65 years). However, there are other submaximal tests commonly used for the elderly and one of them is the 6 min walk test (6MWT). The 6MWT was developed to estimate VO_2_max from a single test (Ebbeling et al. 1991). This test has shown varying results in individuals with cardiopulmonary disorders (*r* = 0.21–0.70, mean *r* = 0.59) (Ross et al. 2010) and does not seem to be a valid test for relatively healthy elderly populations (*r* between estimated and measured VO_2_max for women was not significant, men *r* = 0.8) (Andersson et al. 2011). The 6MWT is easy to perform and has a high correlation with measured VO_2_max for elderly men but not for elderly women, and the test has a high variability with a relative SEE of ~ 27% (Ross et al. 2010). The 5 min pyramid test (5MPT) has a strong correlation to measured VO_2_max in elderly women (*r* = 0.78) and a very strong correlation for elderly men (*r* = 0.98) (Andersson et al. 2011). However, the 5MPT is a maximal test where factors such as motivation and anaerobic capacity may impact results. In comparison, both the EB-test and Å-test are performed submaximally, making them more accessible for populations that are not willing or able to perform maximal effort. The EB method and the Å method had a relative SEE of 11.4% and 20.5%, respectively, in the present study which is better than in the 6MWT.

Although estimated VO_2_max using the EB method agreed with measured VO_2_max in the overall male and female population, estimated VO_2_max for men with low fitness was found to be overestimated. In a previous validation study, including young girls and boys (age 10–15 years), estimated VO_2_max in pre-pubertal boys with similar levels of measured VO_2_max as the low fitness men in the present study was also found to be significantly overestimated (Bjorkman et al. 2018). However, when the EB equation for estimating VO_2_max in women was applied to the pre-pubertal boys in the Bjorkman et al. study, a significantly higher validity and agreement with measured VO_2_max was seen. Hence, we reanalyzed the data from the low fitness men using the EB equation for women. This resulted in a decreased estimation error to non-significant levels [0.04 L min^−1^ (− 0.02–0.09)] and a subsequent higher correlation in all men (*r* = 0.73 using the EB equation for women, compared to *r* = 0.44 using the EB equation for men). However, it was not possible to distinguish the men with low cardiorespiratory fitness using any other variables in the current study other than measured VO_2_max.

We speculate that a decline in testosterone levels with age could affect physiological variables (DeFina et al. 2018; Hosick et al. 2018; Kelsey et al. 2014), which may influence results when using the EB equation for men. Another explanation might be that both pre-pubertal boys and elderly men with low absolute VO_2_max are outside or at the lower end of the VO_2_max range in the EB sample for men in Bjorkman et al. (2016), indicating a more uncertain estimation of VO_2_max in all low fit men (< 2.55 L min^−1^). For clinical practice, the overestimation of elderly men in the present study with low absolute CRF means that elderly men with a general low fitness level and small body stature should be considered at risk of having their VO_2_max overestimated when using the EB-test. Therefore, it would be advantageous for this population to use the EB equation for women.

The Å-test was initially developed for 18–30 year old fairly well-trained individuals (Astrand and Ryhming 1954). Later, an age correction formula was developed to be able to apply the test to a wider range of ages (Astrand 1960). However, even with the age correction formula, many studies have reported that the Å-test still underestimates VO_2_max (Jessup et al. 1977; Jette 1979; Kasch 1984; Hartung et al. 1993), in agreement with the present study on elderly. This could be partly due to the Å-test having been developed with data from both maximal cycle and treadmill tests, since measured VO_2_max tends to be lower when using a cycle ergometer compared to a treadmill (Hermansen et al. 1970). Moreover, in 1960, when the age correction factor was developed, the creators raised a concern that it may underestimate the VO_2_max of older adults by 10% since they displayed lower lactate levels compared to the young adults in the study (Astrand 1960). This led to the belief that the older participants may not have reached their true VO_2_max. Another important factor that could go toward explaining the underestimation of the Å-test in the present study is that the test was designed to be performed on two occasions to eliminate variables such as nervousness and other factors that affect the absolute submaximal HR level on the first test occasion. The EB and the Å-tests both assume a decline in VO_2_max with age, which is usually the case in larger populations (Jackson et al. 2009). A discrepancy between biological and chronological age is a source of error that increases the uncertainty of the EB-test and the Å-test.

In previous studies (Ekblom-Bak et al. 2014; Bjorkman et al. 2016), the difference between estimated and measured VO_2_max using the EB-test was not dependent on maximal HR level. However in the present study, the difference between estimated and measured VO_2_max was found to be dependent on maximal HR for both women and men, when using the EB method. Incidentally, the same result was also seen when using the Å method. The lack of agreement between the current and previous studies is possibly due to the age difference in the sample populations (elderly vs. age mixed), resulting in a higher number of individuals with a low maximal HR in the present study. The present study showed that an individual with low maximal HR has a higher risk of having their VO_2_max overestimated. In previous studies using the EB-test, the difference between estimated and measured VO_2_max was dependent on VO_2_max, as it was in the present study for men, while for women there was a tendency (*p* = 0.051). In other words, elderly men with low measured absolute VO_2_max are at risk of being overestimated and elderly men with high VO_2_max are at risk of being underestimated, when using the EB method. The difference between estimated and measured VO_2_max had a tendency toward a relationship (*p* = 0.059), for the EB method, and was significantly correlated, for the Å method, with self-rated physical activity for women, but not for men. This suggests that the self-rated single item questionnaire may be useful for further strengthening the female, but not the male, algorithms for the estimation of VO_2_max.

## Strengths and limitations

The strength of the study is the modality with which the measured VO_2_max tests were undertaken, i.e. walking or running on a treadmill. It is a modality that most people are familiar with (Lear et al. 1999). A maximal test on a treadmill utilizes greater recruitment of exercising muscle mass than a VO_2_max test performed on a cycle ergometer, where local fatigue in leg musculature could lead up to an 20% lower VO_2_max (Myers et al. 1991). On the other hand, when performing a submaximal VO_2_max test, it is better to use a cycle ergometer because of the low variability in energy expenditure at a certain work rate between individuals (Ekblom and Gjessing 1968).

This study adds to the pool of studies investigating directly measured CRF in an elderly population. Mean VO_2_max in the present study sample was similar to an elderly group in a previous study using the EB-test (Bjorkman et al. 2016). In comparison, a large Norwegian study, where a sample (*n* = 129) of > 70 year olds was tested on a treadmill, reported similar VO_2_max levels (women 1.85 ± 0.35 L min^−1^, men 2.81 ± 0.5 L min^−1^) (Loe et al. 2013) to the present study. Other Nordic studies where VO_2_max was measured directly in the elderly using a cycle ergometer have shown slightly lower VO_2_max values (Andersson et al. 2011; Eriksen et al. 2016). A limitation in the present study could be the self-selection and exclusion criteria resulting in a selected sample of participants. Most likely, this resulted in the present sample being biased toward a higher CRF than would generally be seen in the elderly population in Sweden. The present findings indicate that the EB-test is a good test for the elderly population, but that population-based studies will ultimately be required to ensure generalizability.

## Future perspectives

It has been shown that the EB-test can be used to monitor long-term changes in CRF in an age and gender mixed population (Bjorkman 2017). Future intervention studies are needed to evaluate the ability of the EB-test to also monitor CRF in an elderly population and to identify subtle changes that may result from health promoting interventions such as a physical activity on prescription (Kallings et al. 2008). Another important topic for future research is if the EB-test is affected by certain medicines such as beta-adrenergic blockers and stimulators that affect the function of the cardiorespiratory system. Lastly it would be advantageous to further study the relationship between the EB-test and measured VO_2_max in low fit men and thereafter possibly adjust the EB equation for better VO_2_max estimation.

## Conclusion

The validity of the EB-test in an elderly population was satisfactory both in women and men combined and in women alone but not in men. We found a moderate correlation between the EB method and measured VO_2_max in men; however, there was an overestimation of VO_2_max in men with low fitness. The moderate correlation and overestimation of VO_2_max in low fit men, in contrast to the good correlation and the similarity between estimated and measured VO_2_max in women, could be due to a gender difference in the physiological variables that affect VO_2_max with increasing age. Alternatively, the EB equation for men is unable to correctly estimate VO_2_max in men of all ages with low absolute VO_2_max. The Å method significantly underestimated VO_2_max in both women and men and had a variability that was almost twice that of the EB method. The current study therefore supports using both submaximal methods for population-based studies aiming to evaluate cardiorespiratory fitness as a predictor of health outcomes. On an individual level, the EB method appears suitable for estimating CRF in elderly women, but has insufficient precision for this purpose in elderly men. While the Å method was more accurate than the EB method at identifying the low fit men, its high variability still suggests that it should not be used alone for identifying individuals in need for lifestyle or medical support in elderly populations.

## Electronic supplementary material

Below is the link to the electronic supplementary material.

Supplementary file1 (TIF 7919 kb)

Supplementary file2 (TIF 7919 kb)

Supplementary file3 (TIF 7919 kb)

Supplementary file4 (TIF 7919 kb)

## Abbreviations

Å-test: Åstrand test
CV: Correlation of variance
EB-test: Ekblom-Bak test
HR: Heart rate
LoA: Limits of agreement
PO: Power output/work rate
RER: Respiratory exchange ratio
RPE: Rate of perceived exertion
SEE: Standard error of the estimate
VO_2_max: Maximal oxygen uptake
